# Sarcolipin Exhibits Abundant RNA Transcription and Minimal Protein Expression in Horse Gluteal Muscle

**DOI:** 10.3390/vetsci7040178

**Published:** 2020-11-13

**Authors:** Joseph M. Autry, Christine B. Karim, Sudeep Perumbakkam, Carrie J. Finno, Erica C. McKenzie, David D. Thomas, Stephanie J. Valberg

**Affiliations:** 1 Department of Biochemistry, Molecular Biology, and Biophysics, University of Minnesota, Minneapolis, MN 55455, USA; christine.karim@gmail.com (C.B.K.); ddt@umn.edu (D.D.T.); 2 Department of Large Animal Clinical Sciences, McPhail Equine Performance Center, Michigan State University, East Lansing, MI 48823, USA; perumbakkam@gmail.com; 3 Department of Population Health and Reproduction, University of California, Davis, CA 95616, USA; cjfinno@ucdavis.edu; 4 Department of Clinical Sciences, Oregon State University, Corvallis, OR 97331, USA; erica.mckenzie@oregonstate.edu

**Keywords:** equidae, gene expression profiling, intracellular membranes, long noncoding RNA, peptides, rhabdomyolysis, sarcolipin, protein subunits, sarcoplasmic reticulum calcium-transporting ATPases, western blotting

## Abstract

Ca^2+^ regulation in equine muscle is important for horse performance, yet little is known about this species-specific regulation. We reported recently that horse encode unique gene and protein sequences for the sarcoplasmic reticulum (SR) Ca^2+^-transporting ATPase (SERCA) and the regulatory subunit sarcolipin (SLN). Here we quantified gene transcription and protein expression of SERCA and its inhibitory peptides in horse gluteus, as compared to commonly-studied rabbit skeletal muscle. RNA sequencing and protein immunoblotting determined that horse gluteus expresses the *ATP2A1* gene (SERCA1) as the predominant SR Ca^2+^-ATPase isoform and the *SLN* gene as the most-abundant SERCA inhibitory peptide, as also found in rabbit skeletal muscle. Equine muscle expresses an insignificant level of phospholamban (PLN), another key SERCA inhibitory peptide expressed commonly in a variety of mammalian striated muscles. Surprisingly in horse, the RNA transcript ratio of *SLN*-to-*ATP2A1* is an order of magnitude *higher* than in rabbit, while the corresponding protein expression ratio is an order of magnitude *lower* than in rabbit. Thus, *SLN* is not efficiently translated or maintained as a stable protein in horse muscle, suggesting a non-coding role for supra-abundant *SLN* mRNA. We propose that the lack of SLN and PLN inhibition of SERCA activity in equine muscle is an evolutionary adaptation that potentiates Ca^2+^ cycling and muscle contractility in a prey species domestically selected for speed.

## 1. Introduction

SERCA is a prototypical member of the P-type ion-motive transport ATPase family: central to active membrane transport [1,2]. SERCA Ca^2+^-transporting ATPases are encoded by a family of three genes (*ATP2A1*, *ATP2A2*, and *ATP2A3*) that produce protein isoforms SERCA1 (expressed typically in fast-twitch myofibers), SERCA2 (slow-twitch myofibers, cardiomyocytes, and non-muscle cells), and SERCA3 (smooth muscle cells, platelet cells, and non-muscle cells), respectively. The Ca^2+^ transport activity of SERCA is regulated by a family of single-pass transmembrane peptides in SR, including sarcolipin (SLN) and phospholamban (PLN). SLN and PLN were discovered in the early 1970s [3,4] and have since been characterized as potent regulators of contractility, dependent upon post-translational modifications and protein expression level [5,6,7,8,9]. Recently, five additional peptide regulators of SERCA have been reported in mouse: the inhibitor myoregulin (MRLN) in muscle, the inhibitor small ankyrin 1 (sAnk1) in muscle, the activator dwarf open reading frame (DWORF) in ventricles, the inhibitor endoregulin (ELN) in endothelial and epithelial cells, and the inhibitor “another-regulin” (ALN) in non-muscle tissues [10,11,12,13,14].

The roles of SERCA regulatory peptides in horse SR function are unknown. In mammals such as rabbit, dog, and pig, SLN is the primary regulatory peptide expressed in fast-twitch skeletal muscle [7,15,16,17]. SLN inhibits SERCA activity through multiple enzymatic mechanisms by decreasing maximal velocity (V_max_), by decreasing Ca^2+^ binding affinity (1/K_Ca_), and by decreasing the number of Ca^2+^ ions transported per ATP molecule hydrolyzed (coupling stoichiometry) below the optimal Ca^2+^/ATP coupling ratio of two [18,19,20,21,22,23,24,25,26]. SLN inhibition is relieved in part by phosphorylation and de-acylation [6,27,28], resulting in enhanced SR Ca^2+^ uptake. To optimize myoplasmic Ca^2+^ cycling for muscle performance, SERCA uptake activity must be fine-tuned in balance with Ca^2+^ release activity via the SR Ca^2+^ channel Ryanodine Receptor (RYR). The skeletal muscle isoform RYR1 is regulated by myoplasmic and luminal Ca^2+^ levels, plus Ca^2+^-regulated proteins such as calsequestrin, calmodulin-dependent protein kinase, and calmodulin [29,30,31].

Valberg et al. reported recently the deduced protein sequence and the gene expression level of *SLN*, *MRLN*, and *DWORF* in skeletal muscle of three horse breeds: Thoroughbred, Standardbred, and Quarter Horse [32]. As in other large mammals, the *SLN* gene in horse gluteus is the highest expressed RNA transcript in the family of SERCA regulatory peptides [32]. Phylogenomic analyses of SLN protein sequences across 131 vertebrate species [33] demonstrated that horse SLN has novel deletions and replacements of residues that are predicted to control SERCA regulation and function [7,18,26,32,34]. The horse *SLN* gene encodes a 29-residue peptide [32], in contrast with a consensus 31-residue length for orthologous SLN peptides encoded by 100+ species from five vertebrate classes: mammal, bird, reptile, amphibian, and fish [7,26,32,33]. Uniquely, horse SLN is the only reported ortholog [32,33] missing all four regulatory sites that are common in other species: Ser4 and Thr5 for SLN phosphorylation and relief of SERCA inhibition [6,27]; Cys9 for SLN acylation and relief of SERCA uncoupling [7,28]; and Tyr31 for SERCA interaction, organelle targeting of SLN, and potential luminal nitration of SLN [34,35,36,37,38]; as assessed using residues numbered via the consensus 31-amino acid length, e.g., encoded by rabbit, mouse, and human *SLN* genes [32]. The unique amino acid sequence of horse SLN was also identified in additional *Equus* species such as Zebra and Przewalski horse but not in other species from the perissodactyl order, such as the *Rhinocerotidae* and *Tapiridae* families [32,33]. Thus horses, with natural selection as prey animals and subsequent selective breeding for performance, may have developed highly adapted mechanisms for Ca^2+^ transport regulation including control of SERCA activity by SLN.

Horses are susceptible to exertional rhabdomyolysis, with a 3% incidence reported across breeds, including a 4% incidence in endurance Arabians and a 7–10% incidence specific to racehorses [39,40,41]. The molecular mechanism that causes the high susceptibility of horse to rhabdomyolysis is unknown; however, a common pathway for rhabdomyolysis seems to be associated with aberrant SR Ca^2+^ regulation [42]. Dantrolene, which inhibits Ca^2+^ release through RYR1 in SR, is a muscle relaxant that is used often to treat rhabdomyolysis in horses [43,44,45], thereby supporting further the Ca^2+^–linkage hypothesis. We propose that further investigation of Ca^2+^ cycling in horse muscle will provide insights into unique adaptations for athletic prowess, plus identify molecular targets for potential treatment of rhabdomyolysis in horse breeds.

The goal of this study was to compare gene and protein expression of SERCA, SLN, and PLN in healthy horse muscle, with comparison to the commonly studied model of rabbit skeletal muscle. Our research identified unique steady-state levels of RNA transcription and protein expression for SLN, and these findings were interpreted in light of the high susceptibility of horses to exertional rhabdomyolysis. We propose that SERCA activity and SLN regulation in horse SR differs from the SERCA‒SLN system in rabbit SR, based on unique sequences and expression levels of horse orthologs. We hypothesize that comparative studies of gene expression and biochemical regulation of SR enzymes will increase the broader understanding of selective adaptation of horse and human muscle, with a specific focus towards performance and disease.

## 2. Materials and Methods

### 2.1. Sequences

The Enzyme Commission number (EC) for the SERCA Ca^2+^-transporting ATPase is EC 7.2.2.10 in the IUBMB Enzyme Database. The enzyme for an EC number can be identified through the ExplorEnz utility [46].

The GenBank accession code for cDNA sequences of target proteins and respective species orthologs are as follows: (1) SERCA1a: horse XM_001502262.6, rabbit ABW96358.1, mouse BC036292.1 [47], and human NM_004320.4 [48]. (2) SLN: horse [32], rabbit U96091.1 [49], mouse NM_025540.2, and human U96094.1 [49]. (3) PLN: horse [32], rabbit Y00761.1 [50], human M63603.1 [51], and dog NM_001003332.1 [52]. (4) MRLN: horse [32] and human NM_001304732.2 [10]. (5) DWORF: horse [32] and mouse NM_001369305.1 [12]. (6) GP: horse muscle NP_001138725.1, rabbit muscle NP_001075653.1, and human muscle AH002957.2. The cDNA sequence for an accession code can be identified through the NCBI GenBank utility [53].

### 2.2. Animals and Samples

Six healthy endurance Arabian horses (13.0 ± 6.2 yr, with four castrated males and two females) were used for transcriptomic analyses. Percutaneous needle biopsies had previously been obtained from a standardized site on the gluteus medius muscle [54]. Owner consent was obtained with IACUC approval from Oregon State University with ACUP # 4480.

Protein immunoblotting required 200 g of muscle in order to isolate highly-purified SR membranes [55,56,57,58]. This necessitated euthanasia, and therefore horses donated to the University of Minnesota due to severe orthopedic lameness, were used for biochemical studies of muscle SR proteins [58]. Muscle samples were obtained from four horses aged 10–18 years: three castrated males (two Quarter Horses and one Thoroughbred) and one female (Quarter Horse). Horse owners provided written consent for obtaining samples for this research, with IACUC protocol # 1511-33199A.

For SR isolation from rabbit skeletal muscle, New Zealand white rabbits (junior does less than six months of age) were provided by the Research Animal Resources Facility at the University of Minnesota. Euthanasia of rabbits was performed with appropriate palliative care consistent with American Veterinary Medical Association guideline, with IACUC protocol # 1611-34327A [58].

### 2.3. Muscle Fiber Type Composition

Muscle fiber type composition was determined using a myosin ATPase assay of sections obtained from gluteal muscle that had been frozen in liquid nitrogen-chilled isopentane as previously described [59]. For comparison to horse gluteal muscle, the muscle fiber type composition of seven back and leg muscles from 3‒12 rabbits were compiled from the report by Leberer and Pette [60].

### 2.4. RNA Sequencing of the Horse Muscle Transcriptome

RNA-seq generation of these samples was described previously [59]. In brief, frozen gluteal muscle was ground to powder, and total RNA was extracted. Quantification and quality of RNA were assessed. Samples with an RNA integrity number (RIN) ≥ 7 were used for library preparation and quantification. The library was constructed using a strand-specific polyA^+^ capture protocol (TruSeq Stranded mRNA Kit, Illumina, Inc.). Sequencing was performed using the Illumina HiSeq 2000 genome analyzer (100 base-pair paired-ends) at a targeted 35 million reads/sample. A total of 26,993 transcript sequences from the Ensembl EquCab 2.86 database [61] were used to create the index file and map RNA-seq reads. Samples that passed the quality threshold (Q ≥ 30) were aligned to the horse transcriptome index using Salmon program 0.11.0 [62]. Count data, generated as transcripts per million reads (TPM) by Salmon processing, were used to compile gene expression for each individual sample (N ≥ 5 horses per transcript). The RNA-seq datasets [59] are available in the NCBI Gene Expression Omnibus [63] with GEO accession number GSE104388 [59] and deposited in the Sequence Read Archive (SRA) database with accession number SRP082284.

### 2.5. RNA-seq Quantitation of Gene Expression in Horse and Rabbit Muscle

**Figure 1:**
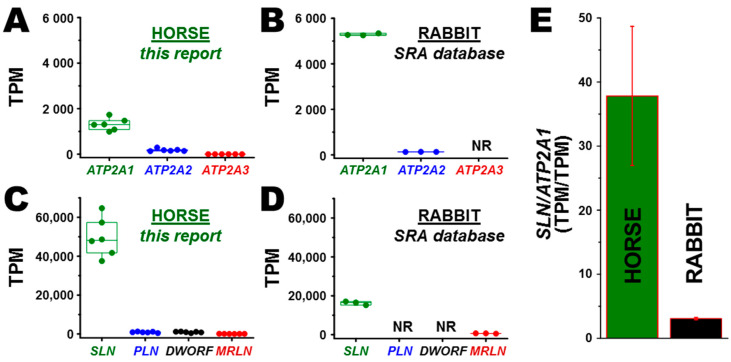
RNA-seq quantitation of gene transcription for calcium regulatory proteins in horse muscle. (**A**–**D**) The number of target transcripts per million reads (TPM) are reported, using a box plot to indicate the median, maximum, minimum, and range (N = 6 Arabian horses and N = 3 rabbits). (**A**,**B**) *ATP2A1* (SERCA1), *ATP2A2* (SERCA2), and *ATP2A3* (SERCA3) in horse gluteus and rabbit muscle, respectively. (**C**,**D**) *SLN*, *PLN*, *DWORF*, and *MRLN* in horse gluteus and rabbit muscle, respectively. (**E**) The relative ratio of transcript/transcript expression (TPM/TPM) of *SLN* to *ATP2A1* in horse gluteus and rabbit muscle, respectively. The expression level of horse genes was mined from RNA-seq data with SRA accession number SRP082284. The expression level of rabbit genes were mined from RNA-seq data reported with SRA accession number SAMN00013649. (**D**) NR indicates “data not reported” for rabbit gene transcripts in the SRA database.

RNA-seq analyses were performed as previously described [59]. Gene expression of seven targets (*ATP2A1*, *ATP2A2*, *ATP2A3*, *SLN*, *PLN*, *DWORF*, and *MRLN*) was extracted from RNA-seq data, normalized based on the length of each transcript and the overall sequencing depth per individual horse, and expressed as transcripts per million (TPM). The values for mean, range, and standard deviation of gene expression are reported as TPM (Figure 1). For comparison, RNA-seq data of gene expression in rabbit muscle (*ATP2A1*, *ATP2A2*, *SLN*, and *MLRN*) were mined from SRA accession number SAMN00013649^3^ (rabbit species *Oryctolagus cuniculus*) (Figure 1).

### 2.6. Purification of SR Vesicles from Horse Gluteal Muscle

Following humane euthanasia of donated horses, one middle gluteal muscle per horse was harvested, and mechanical disruption and differential centrifugation were used to isolate SR vesicles [58]. Horse SR vesicles are defined as the centrifugal pellet isolated at 10,000× *g_max_* for 20 min (the “10KP” cellular fraction), which was preceded by two clarification spins at 4000× *g_max_* for 20 min (see our horse SR protocol flow-chart reported as Figure 1 in [58]). The protein concentration of horse SR vesicles was determined by the BCA assay (Pierce Biotechnology).

### 2.7. Purification of SR Vesicles from Rabbit Skeletal Muscle

Following humane euthanasia of rabbits, the back and leg muscles per rabbit were harvested, and mechanical disruption and differential centrifugation were used to isolate SR vesicles [58,64]. Rabbit SR vesicles are defined as the centrifugal pellet isolated at 23,000× *g_max_* for 60 min (the “23KP” cellular fraction), which was preceded by three clarification spins: 4000× *g_max_* for 20 min, 4000× *g_max_* for 20 min, and 12,000× *g_max_* for 20 min (see the rabbit SR protocol flow-chart reported as Figure S1 in [58]). The protein concentration of rabbit SR vesicles was determined by the BCA assay (Pierce Biotechnology).

### 2.8. Synthesis of SLN Peptide Orthologs for Quantitative Immunoblotting

Horse SLN (29 residues), rabbit SLN (31 residues), and human SLN (31 residues) peptides were synthesized by New England Peptides, Inc., using Fmoc solid-phase chemistry at 50-mg crude scale (16 µmol). Quality assessment of peptide synthesis was validated using high-performance liquid chromatography (HPLC) and mass spectrometry. The three SLN peptides were synthesized with an acetylated N-terminus and an amidated C-terminus. Synthetic SLN peptides were further purified in-house by HPLC [65]. The concentration of synthetic SLN peptides was determined by amino acid analysis, BCA assay, and densitometry of Coomassie blue-stained gels [34,66]. Laemmli-type SDS-PAGE gels were purchased from Bio-Rad Laboratories, Inc.

### 2.9. Quantitative Immunoblotting of Muscle Proteins

Immunoblotting was performed as previously described [55,56,67,68]. Proteins separated by SDS-PAGE were transferred to a PVDF membrane (0.2-µm pore Immobilon-FL) using a solution of 25 mM Tris, 192 mM glycine, pH 8.3. PVDF blots were blocked using Odyssey-TBS blocking buffer (LI-COR Biosciences, Inc., Lincoln, NE, USA). Primary antibodies are described below. Primary antibodies were used typically at 1:1000 dilution, with incubation for 16–18 h at 4 °C. Secondary antibodies (fluorescently-labeled) were used at 1:15,000 dilution, with incubation for 20 min at 4 °C. After incubation with primary or secondary antibody, blots were washed three times with Tris-buffered saline (pH 7.4) with 0.05% Tween-20, and then once with Tris-buffered saline (pH 7.4) without Tween-20. Sandwich-immunolabeling of the target protein via primary plus secondary antibodies was detected using an LI-COR laser scanner in near-infrared fluorescence mode equipped with Odyssey acquisition software, and bands were quantified using Image Studio Lite software (LI-COR Biosciences, Inc.) on a local PC computer (see next section). 

### 2.10. Secondary Immunolabeling and Target Quantitation Using Fluorescent Anti-IgG Antibodies

Secondary antibodies labeled with near-infrared fluorophore (700 nm or 800 nm emission) were purchased from LI-COR Biosciences, including affinity-purified goat antisera against mouse IgG (goat anti-mouse pAb) and affinity-purified goat antisera against rabbit IgG (goat anti-rabbit pAb). Secondary antibodies were used at 1:15,000 dilution (20 min at 4 °C) for sandwich-labeling of target protein following primary antibody incubation. Fluorescent bands were detected using an Odyssey laser scanner in near-infrared mode and quantified using Image Studio Lite software (LI-COR Biosciences, Inc.). For quantitative comparison of band intensities via Image Studio Lite, a box was drawn around the band of interest, and the fluorescence intensity was extracted using LI-COR software. For each immunoblot, the background intensity was determined by drawing a same-sized box (as target band) in a protein-free lane, and this background intensity was subtracted from the target protein signal. For a target protein that showed multiple oligomeric species, the intensity of each monomeric and oligomeric band was quantitated and then added together for a sum total of target per sample. For quantitative standard, a synthetic peptide of SLN or PLN were utilized as appropriate, with the peptide standard loaded in multiple amounts (low nanogram range), followed by linear-regression analysis of immunoblot intensity versus protein load [55,56,57,68].

### 2.11. Custom Anti-Horse-SLN Polyclonal Antibody GS3379

**Figure 2:**
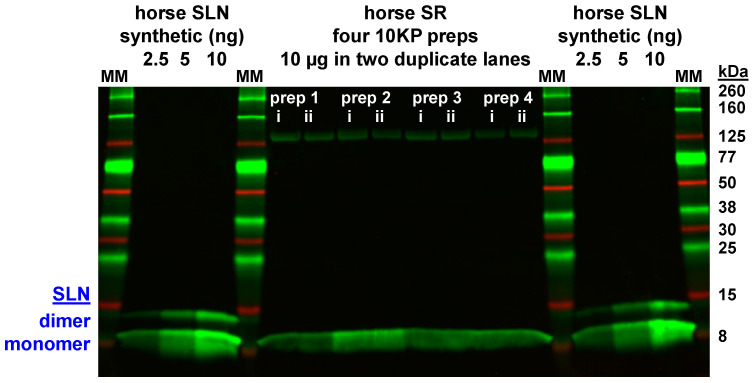
Immunoblot analysis using custom anti-horse-SLN pAb GS3379 detects minimal expression of SLN protein in horse SR vesicles. The primary antibody was the custom anti-horse-SLN pAb GS3379, with immunogen comprising horse residues ^1^MEWRRE^6^. Samples were electrophoresed through a 4–20% Laemmli gel. Horse SR vesicles were assayed (N = 4 preps; 10 µg protein per lane; each prep run in duplicate lanes). Synthetic horse SLN was used as a quantitative standard (2.5, 5, or 10 ng/lane) in duplicate sets on left and right sides on the gel. Immunoblotting was performed using pAb GS3379 (primary) and goat anti-rabbit-IgG pAb labeled with 800-nm fluorophore (secondary). Immunolabeling was quantified using the LI-COR laser scanner system. Protein gel markers (kDa) are indicated on the right. Two putative molecular species of SLN on SDS-PAGE (monomer and dimer) are indicated by blue text on the left.

**Figure 3:**
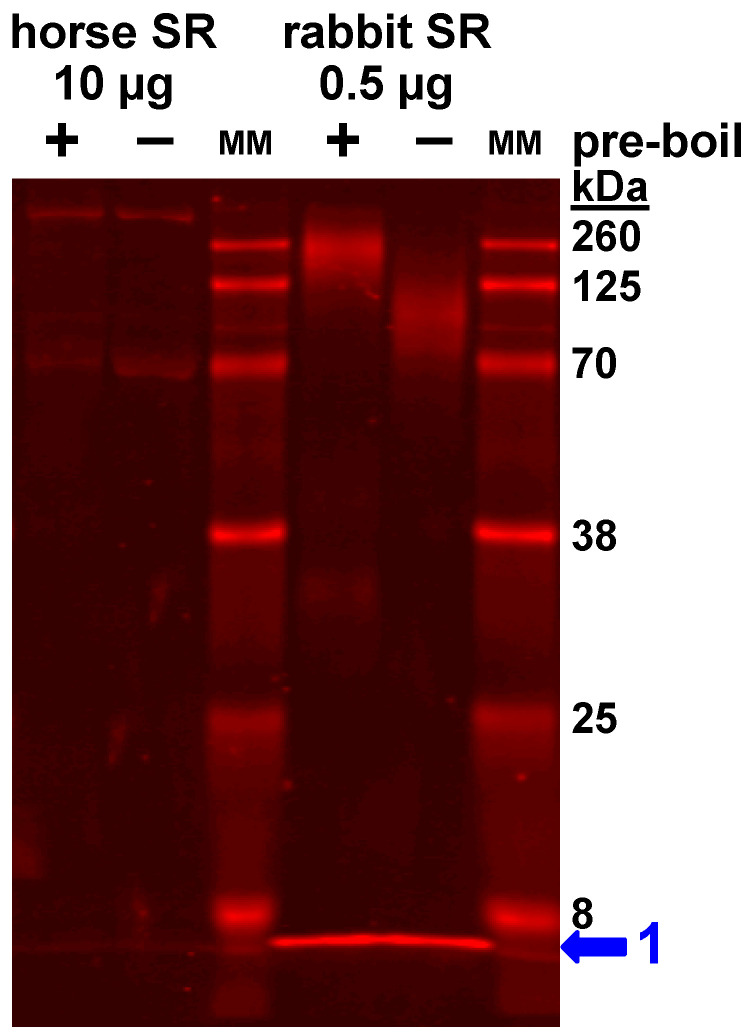
Immunoblot analysis using commercial anti-rabbit/mouse/human-SLN pAb ABT13 identifies SLN protein expression in rabbit SR vesicles, but not in horse SR. The primary antibody was the commercial anti-SLN pAb ABT13, with immunogen comprising rabbit/mouse/human SLN residues ^27^RSYQY^31^, whereas horse SLN encodes ^26^RSYQ^29^. Samples were electrophoresed through a 10% Laemmli gel. Horse SR vesicles were loaded at 10 µg protein per lane (left), and rabbit SR vesicles were loaded at 0.5 µg protein per lane (right). Immunoblotting was performed using pAb ABT13 (primary) and goat anti-rabbit-IgG pAb labeled with 680-nm fluorophore (secondary). Immunolabeling was quantified using the LI-COR laser scanner system. Prior to electrophoresis, one sample of each SR prep was heated at 100 °C for 2 min in Laemmli sample buffer (+ pre-boil). Protein gel markers (kDa) are indicated on the right. The putative monomeric form of SLN on SDS-PAGE is indicated by the blue arrow with number 1 on the right.

Due to sequence divergence of horse SLN at the N-and C-termini compared to rabbit, mouse, and human orthologs, we designed and purchased an anti-horse-SLN polyclonal antibody from Genscript (pAb GS3379). The immunogen was a 6-residue N-terminal peptide of horse SLN (Q^1^MEWRRE^6^C), with an added ^−1^Gln residue (to mimic an acetylated N-terminus) along with an added ^+7^Cys residue (to serve as a C-terminal linker). The horse N-terminal SLN peptide was conjugated to keyhole limpet hemocyanin (KLH) as the carrier protein for rabbit immunization. The anti-horse-SLN pAb GS3379 was purified from rabbit serum using affinity purification on the same horse-SLN-peptide coupled as an affinity-column ligand. Antibody generation, affinity purification, and titer determination were performed by Genscript. Here pAb GS3379 was used for primary labeling at 1:1000 dilution (0.4 µg/mL). pAb GS3379 was validated using synthetic horse SLN as a quantitative immunoblot standard (Figure 2). It is readily apparent that SR vesicles from horse gluteal muscle express a minimal amount of SLN (Figure 2 and Figure 3, Figure S2), at sub-stoichiometric amounts compared to SERCA. The low expression of SLN in horse gluteal muscle SR was previously demonstrated in preliminary immunblot experiments during the development of the isolation procedure for horse SR vesicles [69], as utilized in this publication.

### 2.12. Custom C-Terminal Anti-Rabbit/Mouse/Human-SLN Polyclonal Antibody PFD-1

Dr. Patrick F. Desmond and Professor Robert J. Bloch, from the University of Maryland School of Medicine, provided a custom-generated anti-SLN pAb [70]. Two immunogens were used for rabbit immunization: a base 7-residue C-terminal peptide conserved in rabbit/mouse/human SLN with alternate variations in SLN immunogenic peptides at N-and C-termini positions (acetyl-^25^LVRSYQY^31^-amide and C-aminohexanoic acid-^25^LVRSYQY^31^-OH). Both SLN peptides were individually conjugated to BSA. Antibody generation and affinity purification were performed by 21st Century Biochemicals, Inc. The anti-rabbit/mouse/human-SLN pAb PFD-1 was validated for immunoblotting using mouse skeletal muscle homogenate and recombinant mouse SLN [70]. Horse SLN encodes ^24^LVRSYQ^29^ (i.e., horse SLN is missing one residue of the seven-residue immunogen: the terminal Tyr residue comprising the pAb PFD-1 epitope). Here pAb PFD-1 was used for primary labeling at 1:1000 dilution. Although horse SLN encodes six of seven residues in the immunogen peptide sequence, horse SR vesicles show significantly lower labeling than rabbit SR vesicles by pAb PFD-1 (Figure S2).

### 2.13. Commercial Anti-SLN Polyclonal Antibodies

Two anti-SLN commercial antibodies were purchased. One commercial anti-SLN antibody (ABT13) was raised against the mostly conserved C-terminus of SLN. One commercial anti-SLN antibody (pAb 18395-1-AP) was raised against human SLN (N-terminal peptide or full-length protein). Immunoblot conditions and validation reports for commercial anti-SLN antibodies are described as follows:

(1) Anti-SLN ABT13 is an affinity-purified rabbit pAb (Millipore Sigma). The immunogen was C-terminal residues ^26^VRSYQY^31^ of rabbit/mouse/human SLN as a sulfolink-peptide (CGG-^26^VRSYQY^31^) conjugated to KLH. Horse SLN encodes ^26^VRSYQ^30^ (i.e., missing the terminal Y31 of the rabbit/mouse/human SLN immunogen). Millipore Sigma reports that ABT13 reacts with rabbit, mouse, and human SLN on immunoblot at 1.5 µg/mL pAb, as corroborated here for rabbit SLN using 0.5 µg/mL pAb ABT13 (1:1000 dilution) and fluorescent secondary antibody detection (Figure 3). pAb ABT13 has been validated for SLN immunoblotting using human right atrium homogenate [71] and mouse soleus and diaphragm homogenates [72]. Here pAb ABT13 was used for primary labeling at 1:1000 dilution, with positive detection of SLN in rabbit SR vesicle, plus sunthetic rabbit and human SLN (Figure 3).

(2) Anti-human-SLN 18395-1-AP is an affinity-purified rabbit pAb (Proteintech Group, Inc.). The immunogen was human SLN (residues 1–31) conjugated as a fusion protein to glutathione S-transferase (GST). Compared to human SLN protein, horse SLN is 77% identical (5 sequence variations and 2 deletions), and rabbit SLN is 81% identical (6 sequence variations) [32,33]. Proteintech Group reports that pAb 18395-1-AP reacts with SLN in human and mouse heart homogenates on immunoblot using antibody concertation of 1.6 µg/mL and 0.5 µg/mL pAb, respectively. pAb 18395-1-AP has been validated on SLN immunoblot using mouse heart homogenate [73]. Here pAb 18395-1-AP was used for primary labeling at 1:1000 dilution, a condition where pAb 18395-1-AP did not detect rabbit or horse SLN but instead showed (non-specific?) binding to other proteins in rabbit and horse SR (Figure S1).

### 2.14. Anti-PLN Monoclonal Antibody 2D12

**Figure 4:**
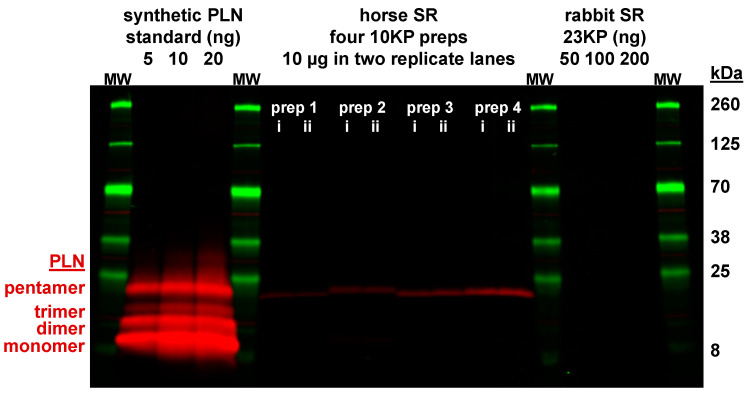
Immunoblot analysis using anti-universal-PLN mAb 2D12 detects minimal expression of PLN protein in horse and rabbit SR vesicles. The primary antibody was the commercial anti-PLN mAb 2D12 with epitope ^7^LTRSAAIR^13^, a sequence which is identical in the three PLN orthologs: horse, horse, and rabbit. Samples were electrophoresed through a 4–20% Laemmli gel. Horse SR vesicles (N = 4 preps; 10 µg protein per lane; each prep run in duplicate lanes) and rabbit SR vesicles (50, 100, 200 ng) were assayed. Synthetic human PLN (5, 10, 20 ng) was used as a quantitative standard. Immunoblotting was performed using mAb 2D12 (primary) and goat anti-mouse-IgG pAb labeled with a 680-nm fluorophore (secondary). Immunolabeling was quantified using the LI-COR laser scanner system. Protein gel markers (kDa) are indicated on the right. The proposed molecular species of PLN (monomer, dimer, trimer, and pentamer) on SDS-PAGE are indicated on the left.

Anti-PLN antibody 2D12 is a mouse mAb (IgG2a isotype) generated by Dr. Larry R. Jones [74]. The immunogen of mAb 2D12 was residues 2–25 of dog PLN conjugated to thyroglobulin using glutaraldehyde. The epitope for mAb 2D12 is residues ^7^LTRSAIR^13^ of dog PLN [75], a sequence which is identical for horse and rabbit PLN [32]. mAb 2D12 (ab2685) was purchased from Abcam, P.L.C., as a stock solution of Ascites fluid diluted to 1 mg/mL mAb protein in PBS. Abcam reports that mAb 2D12 reacts on immunoblot with PLN peptides expressed in rabbit, mouse, human, pig, sheep, chicken, guinea pig, and dog tissues. mAb 2D12 has been validated for quantitative immunoblotting using multiple types of PLN samples: native and recombinant dog PLN, native mouse PLN, native human PLN, synthetic human PLN, and synthetic pig PLN [55,56,57,67,68,74,76,77]. Here anti-PLN mAb 2D12 was used for primary labeling at 1:1000 dilution (1 µg/mL), with successful detection of synthetic human PLN and horse PLN, as utilized as standards for quantitative immunoblotting (Figure 4). It is readily apparent that SR vesicles from horse gluteal muscle express an insignificant level of PLN.

### 2.15. Experimental Design, Statistical Analysis, and Data Presentation

Biochemical assays were performed using independent SR vesicle preparations from N = 4 horses. Data analyses were performed using Microsoft Excel. Data graphs were generated using OriginLab 9.2 (Northampton, MA, USA). Scientists were not blinded to sample identity during data acquisition or analysis. Western blot analysis, i.e., signal intensity, was quantified using the LI-COR laser scanner system, via the associated Odyssey acquisition and Image Studio Lite analysis programs, provided by LI-COR, Inc. Data acquired in this study are presented in mean ± standard deviation.

## 3. Results

### 3.1. Muscle Fiber Type Composition

The middle gluteal muscle of six healthy Arabian horses had a composition of type 1 myofibers with 17 ± 2% (slow-twitch), plus type 2A myofibers with 52 ± 8% (fast-twitch glucoxidative) and type 2X (fast-twitch) with 31 ± 8%, thereby expressing a 4.9 ± 0.9 fold greater abundance of fast-twitch myofibers [59]. For comparison, a compilation of seven types of rabbit white back and leg muscles shows a predominance of fast-twitch myocytes (type 2A with 21 ± 7.6% and type 2B with 76 ± 8.7%) versus slow-twitch myocytes (type I = 3.1 ± 3.1%) [60]. Although horse gluteal muscle has a greater percentage of type 1 slow-twitch myofibers than rabbit skeletal muscle (*p* < 0.0001), the predominance of fast-twitch myofibers in horse gluteus and rabbit muscle allows suitable comparison of SERCA1 and its control by peptide regulators.

### 3.2. Transcription of ATP2A Genes Encoding SERCA Proteins

Transcriptomic analysis identified the *ATP2A1* gene encoding SERCA1 as the predominant SERCA isoform expressed in the middle gluteal muscle of horse at 7.3 ± 2.8 fold greater level than the *ATP2A2* gene encoding SERCA2 (Figure 1A). The middle gluteal muscle of horse showed insignificant expression of the horse *ATP2A3* gene, which is transcribed at a level comprising ~ 0.04% of total transcripts from the *ATP2A* gene family (TPM/TPM) (Figure 1A). In rabbit, mined RNA-seq data demonstrated that the *ATP2A1* gene (SERCA1) is expressed at 41 ± 1.0 fold greater level than the *ATP2A2* gene (SERCA2) (Figure 1B). Thus, both horse gluteal muscle and rabbit skeletal muscle express predominantly the *ATP2A1* gene, thereby providing a valid platform for comparative transcriptomic and biochemical analyses of SR function in these predominantly fast-twitch muscles.

### 3.3. Transcription of SERCA Regulatory Peptide Genes: SLN, PLN, MRLN, and DWORF

*SLN* is the predominant regulatory peptide transcribed in horse gluteal muscle, with >55-fold greater expression than *PLN* and *DWORF* and 780 ± 420 fold greater expression than *MRLN* (Figure 1C). Transcription of the *SLN* gene is 38 ± 11-fold greater than *ATP2A1* (SERCA1), thereby demonstrating that the *SLN* gene is highly expressed in the horse gluteus muscle (Figure 1A,C,E).

A compilation of reported data (SRA database) from three laboratory rabbits indicated that the *SLN* gene is the predominant regulatory peptide transcribed in rabbit skeletal muscle, with 26 ± 4.3-fold greater gene expression than *MRLN* (Figure 1D). When comparing the relative ratio of gene expression of *SLN* to the sum of *ATP2A* genes, rabbit muscle shows a transcription ratio of 3.1 ± 0.19 for *SLN*-to-*ATP2A1* (TPM/TPM), a ratio which is ~12-fold lower than the *SLN*-to-*ATP2A1* ratio in horse muscle (Figure 1B,D,E). Thus, our RNA-seq data is consistent with previous qRT-PCR studies indicating that *SLN* RNA is the predominant regulatory gene transcribed in fast-twitch muscle of the horse, similar to other non-rodent mammals such as rabbit and pig [15,32]. *MRLN* is reported to be the primary regulatory peptide gene expressed in mouse skeletal muscle [10].

### 3.4. Expression of the SLN Peptide Relative to SERCA Protein in Horse SR vesicles, as Detected by Multiple Anti-SLN Antibodies

To determine if SLN peptide expression correlates with *SLN* gene transcription, immunoblotting was used to quantitate the expression level of native SLN peptide in horse muscle (Figure 2 and Figure 3, Figures S1 and S2). Due to the unique amino acid sequence of horse SLN [32], a custom anti-horse-SLN pAb was ordered (GS3379) using horse SLN residues ^1^MEWRRE^6^ (N-terminal peptide) as immunogen. In addition, a full-length equine SLN peptide (amino acids 1–29) was synthesized as a positive control for quantitative immunoblotting [55,56]. On immunoblots, anti-horse-SLN pAb GS3379 detected synthetic horse SLN loaded at 2.5‒10 ng SLN per lane (Figure 2), thereby validating pAb GS3379 as an avid antibody for detection of horse SLN.

Per immunoblotting of horse SR vesicles, anti-horse-SLN pAb GS3379 showed a small amount of detection of native SLN expression in horse SR vesicles (10KP fraction), when horse SR vesicles were when loaded at 10 µg SR protein per lane, albeit at a lower intensity level compared to 10 ng of synthetic horse SLN (Figure 2). Thus, pAb GS3379 indicates that the SLN peptides comprises < 0.1% of the total protein in horse SR vesicles. In comparison to synthetic horse SLN standard, immunoblot analysis of four horse SR preps using pAb GS3379 determined that native SLN is expressed at 0.16 ± 0.015 nmol SLN/mg SR protein (Figure 2). The expression level of SERCA in horse SR is estimated at ~2.1 nmol SERCA/mg SR protein, per content assessed by Coomassie densitometry and immunoblotting [58], using rabbit SR vesicles as a relative standard with 5.0‒6.4 nmol SERCA/mg SR protein [78,79,80]. Thus, immunoblotting using the anti-horse-SLN pAb 3379, with synthetic horse SLN peptide as quantitative standard, determined that SR vesicles from horse gluteal muscle express a minor stoichiometric ratio of ~0.06 SLN/SERCA (mol/mol), i.e., there are ~16 SERCA molecules per available SLN subunit. Since the binding affinity of SLN‒SLN self-association is similar to SLN‒SERCA regulatory complex formation [81,82], thereby depleting the availability of inhibitory SLN monomers, we conclude that horse SR expresses a large majority of SERCA as SLN-free pumps.

On immunoblots, the small amount of horse SLN in native SR vesicles migrates as a monomer on SDS-PAGE (Figure 2, Figure 3 and Figure S2), as is typically found for rabbit SLN in SR vesicles, and also other SLN orthologs in species-specific SR vesicles, e.g., mouse, dog, and pig [15,17,83,84]. On the other hand, purified SLN (native, recombinant, or synthetic) on SDS-PAGE migrates occasionally as a mix of oligomeric species: abundantly-populated monomers with a variable amount of dimers, and sometimes with lowly-populated trimers and/or tetramers [18,34,85,86,87] (Figure 2, Figure 3 and Figure S2). Thus, denaturing SDS-PAGE maintains a variable amount of self-association for purified SLN.

Further attempts to detect SLN in horse and rabbit muscle utilized additional commercial and custom-made anti-SLN antibodies. Horse SLN shows sequence divergence and a 1-residue deletion (ΔS4) at the N-terminus, plus a 1-residue deletion (ΔY30/31) at the C-terminus. The commercial anti-rabbit/mouse/human-SLN pAb ABT13, with immunogen comprising the consensus C-terminus of SLN (residues ^27^RSYQY^31^), successfully detected SLN expression in rabbit SR (0.5 µg SR protein/lane), but pAb ABT13 did not detect SLN in horse SR (10 µg SR protein/lane) (Figure 3). The custom anti-rabbit/mouse/human-SLN pAb PFD-1 generated by Desmond et al., with immunogen comprising a 7-residue stretch of the consensus C-terminus of SLN (residues ^25^LVRSYQY^31^) [70], successfully detected rabbit SLN expression in SR vesicles (0.1 µg SR protein/lane) and also synthetic rabbit SLN (0.01 µg SLN/lane), but pAb PFD-1 did not detect horse SLN in gluteal SR vesicles (10 µg SR protein/lane) nor synthetic horse SLN (25 ng SLN/lane) (Figure S2). The commercial pAb 18-395-1-AP, with immunogen comprising human SLN (residues 1–31), did not detect SLN in rabbit SR (0.5 µg SR protein/lane) or horse SR (10 µg SR protein/lane) (Figure S1). In our assessment, the custom anti-horse-SLN pAb GS3379 gives suitable and reproducible immunoblot results for detecting horse SLN. Furthermore, the commercial anti-rabbit/mouse/human-SLN pAb ABT13 and the custom anti-rabbit/mouse/human-SLN pAb PFD-1, provided by Bloch lab [11,70], both give suitable and reproducible immunoblot results for detecting rabbit SLN. The use of multiple custom and commercial antibodies showed minimal-to-no expression of the SLN peptide in horse SR vesicles.

### 3.5. Correlation of Gene and Protein Expression of SLN in Horse and Rabbit Muscle

When correlating the gene expression of *SLN* versus the protein expression of SLN in horse muscle (normalized per *ATP2A1* and SERCA1, respectively), we determined that the high level of *SLN* RNA, with *SLN*/*ATP2A1* = 38 ± 11 (TPM/TPM) (Figure 1E), produces an insignificant amount of SLN regulatory peptide, with a protein expression ratio of SLN/SERCA ~0.06 (mol/mol) (Figure 2). Thus, the RNA/RNA expression ratio of *SLN*/*ATP2A* is ~600 fold greater than the protein/protein expression ratio of SLN/SERCA in horse muscle. In comparison, rabbit skeletal muscle shows a relative gene expression ratio of *SLN*/*ATP2A1* = 3.1 ± 0.2 (TPM/TPM) (Figure 1E) and a protein expression ratio of 0.75–1.2 SLN/SERCA (mol/mol) [18,84], which produces a ~3-fold greater level of RNA/RNA vs. protein/protein expression ratio for SLN/SERCA1. Thus, horse muscle lacks a positive correlation of gene and protein expression levels for SLN and SERCA, unlike rabbit muscle.

### 3.6. Horse Gluteus Expresses A Minimal Level of PLN Inhibitory Peptide Compared to SERCA Protein

Horse muscle expresses a minor level of *PLN* transcripts (Figure 1B). To determine if PLN peptide expression correlates with *PLN* gene transcription, immunoblotting was used to quantitate the expression level of native PLN peptide in horse muscle (Figure 4). The commercial anti-PLN mAb 2D12 recognizes an identical N-terminal sequence among horse and rabbit peptides and has been used effectively in multiple types of experiments: immunoblotting, ELISA, and histochemistry, plus functional assays to disrupt PLN inhibition of SERCA in SR vesicles and live cells [74,88,89,90]. Quantitative immunoblotting with anti-PLN mAb 2D12 and a synthetic PLN peptide standard determined that SR vesicles from horse gluteal muscle expresses <0.01 PLN/SERCA (mol/mol) (Figure 4), consistent with previous reports showing low to undetectable level of PLN protein in rabbit muscle [15,91]. The binding affinity of the inhibitory PLN monomer to SERCA is 7‒40-fold lower than the binding affinity of PLN monomers in the competing self-association of PLN into non-inhibitory homo-pentamers [92], thereby reducing greatly the available amount of regulatory PLN monomers (≪0.01 mol/mol SERCA). We conclude that the PLN peptide in SR vesicles from horse gluteal muscle is expressed at an insignificant level compared to the expression level of SERCA protein.

## 4. Discussion

This study is a comparative assessment of gene and protein expression of peptides regulating SERCA Ca^2+^ transport in horse and rabbit muscles, thereby providing fundamental information on Ca^2+^ regulation at the molecular level. We determined that the horse and rabbit skeletal muscles examined were predominantly composed of fast-twitch fibers expressing *ATP2A1* (SERCA1) and therefore were suitable for comparison [15]. We propose that horse muscle utilizes translational and/or degradation mechanism to mediate a low level of SLN protein expression, and that the high level of *SLN* RNA may possess a cellular function besides translation. The generated results provide new insights into Ca^2+^ transport regulation in horse muscle, and a cautionary tale about inferring protein expression level from gene expression analysis.

### 4.1. Gel analysis for Oligomerization of Horse and Rabbit SLN

On immunoblots, the minimal amount of horse SLN in native SR vesicles migrated as a monomer on SDS-PAGE (Figure 2, Figure 3 and Figure S2), as is typically found for rabbit SLN expressed in SR vesicles, and also other orthologs (mouse, dog, and pig) in respective SR vesicles [15,17,83,84]. Conversely, purified SLN (native, recombinant, or synthetic) on SDS-PAGE migrates often as a mix of oligomeric species: abundantly-populated monomers, a variable amount of dimers, and sometimes with lowly-populated trimers and/or tetramers [18,34,85,86,87] (Figure 2 and Figure S2). Higher-order oligomers of SLN in SR are proposed to play key roles in SERCA regulation, as SLN oligomerization competes directly with the binding of inhibitory SLN monomers to SERCA [34,81,86,93,94]. A similar competition of peptide–pump interactions has been well characterized for the PLN–SERCA system, whereby PLN self-association into pentamers competes with the assembly of a PLN monomer and SERCA monomer into an inhibitory hetero–dimeric complex, with the avidity depending on a competing binding equilibria that are dynamically controlled by PLN phosphorylation and SERCA Ca^2+^ binding [67,95,96]. Oligomerization of synthetic horse SLN on SDS-PAGE suggests that self-association of horse SLN plays a role in the availability of SLN monomers for inhibition of horse SERCA. Binding of SLN and SERCA in a multimeric, pea-pod complex (SLN monomer, SLN pentamer, and SERCA monomer) is proposed to be an activating mechanism for the Ca^2+^ activated ATPase function of SERCA [97].

### 4.2. Positive and Negative Correlations of Gene Transcription and Protein Expression of SR Ca^2+^ Transport Regulators in Horse Gluteal Muscle

Transcriptome sequencing is a powerful tool for identifying gene transcription and changes that occur during physiologic or pathologic perturbations. In our study we found that horses have exceptionally high *SLN* transcription relative to other regulatory peptides (*PLN*, *MRLN*, *DWORF*) and SERCA genes (*ATP2A1*, *ATP2A2*, *ATP2A3*). These relative expression levels are consistent with qr-PCR analyses that determined the *SLN* gene (versus *PLN*, *MRLN* or *DWORF*) is the predominant regulatory peptide transcript expressed in the middle gluteal muscle of Thoroughbred horses [32]. mRNA transcript abundance is often used, and can be, an excellent proxy for protein level in terms of whether or not a specific protein is expressed within specific tissues [98,99]. To infer more exact cellular concentration of proteins is imprecise because on average protein level correlates with the abundance of corresponding mRNA with a squared Pearson correlation coefficient of 0.40 [100]. Stronger positive correlation for Ca^2+^ regulatory transcript abundance and protein expression, however, has been shown in a mouse microarray-proteomic study that included calsequestrin (*CASQ2* r = 0.999893) and the sodium/potassium-transporting ATPase catalytic subunit (*ATP1A1* r = 0.901647) [101]. Vangheluwe et al. found that *SLN* mRNA expression in skeletal muscle correlates positively to SLN peptide expression in mouse, rat, rabbit, and pig skeletal muscle [15]. Previous work has shown also that SLN peptide expression correlates with *SLN* mRNA transcription in mouse leg muscle [102].

To detect horse SLN in horse gluteal muscle, we tested commercial and custom-made antibodies. The pAb ABT13, with immunogen the consensus C-terminus of SLN (^27^RSYQY^31^), showed little to no immunoreactivity for SLN in horse SR vesicles, yet pAb ABT13 identified successfully SLN in rabbit SR vesicles (Figure 3). Our custom anti-horse-SLN pAb GS3379 was successful in detecting synthetic horse SLN; this custom pAb detected minimal levels of SLN expressed in horse SR vesicles (Figure 2). Thus, correlating *SLN* gene expression with SLN protein expression, we found that the high level of *SLN* RNA transcripts in horse muscle do not result in significant SLN protein production. The protein expression ratio of SLN/SERCA was ~0.06 (mol/mol) for horse compared to 0.75 or 1.2 SLN per SERCA (mol/mol) rabbit skeletal muscle [18,84]. Thus, it was quite remarkable that, in spite of mRNA abundance, our immunoblotting results demonstrated an insignificant expression of SLN in horse SR compared to SERCA.

In addition, gene and protein expression of the SERCA inhibitor PLN was negligible in horse muscle with a ratio of <0.01 PLN/SERCA (mol/mol) (Figure 4). Also, minimal gene transcription was found for *MRLN* and *DWORF* (Figure 1). Thus, our results suggest that horse gluteus is a native adult mammalian muscle that shows insignificant-to-undetectable expression of the most-common regulatory peptides of SERCA (e.g., SLN, PLN, and MLRN). We acknowledge that additional research is needed to support this claim.

### 4.3. Is There a Mammalian Striated Muscle Reported to Expresses SERCA in the Absence of Regulatory Transmembrane Peptide?

The long-held traditional view on Ca^2+^ regulation in striated muscles (skeletal and cardiac) has been that in each specific myocyte type, SERCA is co-expressed with one specific regulatory transmembrane peptide (either SLN or PLN or MLRN) to produce a reversible inhibitory interaction, forming an heterodimeric complex whereby SERCA activity is inhibited until post-translational modification of the regulatory peptide subunit occurs (for review, see Primeau et al. [103]). More recently, there have been reports of three-way co-expression of SERCA, SLN, and PLN together, as assessed at the single-cell level, in human leg postural muscle (vastus lateralis) and Takotsubo cardiomyopathy patients (left ventricle), with enhanced SERCA inhibition [16,104]. These results support MacLennan’s seminal report of “super-inhibitory” trimeric complexes comprising one catalytic pump (SERCA) and two inhibitory subunits (SLN and PLN), proposed to be expressed in native muscle and heart [105,106], as initially assessed via cell culture and mouse models of epitope-tagged peptide regulators. As additional peptide regulators of SERCA continue to be identified, the gene/peptide family has been termed “regulins” [13,82,103,107,108].

It is becoming clear that SERCA and its regulatory peptides are expressed broadly in tissue-specific patterns, with concomitant single and dual physiological coupling to SERCA [16,70,105,109]. In mouse slow-twitch muscle, genetic knock-out of SLN expression results in enhanced ATP-dependent Ca^2+^ uptake by SERCA [110]. In patients with atrial fibrillation, or heart failure with preserved ejection fraction, decreased expression of SLN correlates with increased Ca^2+^ uptake in atrial homogenates; however, it is unknown whether decreased SLN inhibition and subsequent SERCA activation are *compensatory* or *causative* in disease progression [111,112]. It is possible that the *Equus* species evolved a genetic short-cut for speed to evade predators whereby a unique SLN peptide sequence and minimal expression level leaves SERCA function uninhibited, thereby providing rapid relaxation and enhanced contractility for the next muscle contraction. We identified a surprising disconnection between the supra-abundant transcription of *SLN* mRNA from significant expression of the SLN protein. We propose that horse muscle utilizes translational and/or degradation mechanisms to mediate a low basal level of SLN protein, and that the high level of *SLN* RNA may possess a cellular function besides translation.

### 4.4. Does the SLN Gene Transcript Act as A Functional Long Non-Coding RNA for Controlling Contractility of Horse Gluteal Muscle?

Transcriptional regulation is the primary mechanism for the control of eukaryotic protein expression, yet throttling of protein translation from mRNA is also common, including effects by encumbered 5′ noncoding sequences, regulation by micro-RNAs, and accessory proteins binding to mRNA or ribosomes [113]. At first, our unique detection of the dissociation between *SLN* RNA and SLN protein levels in horse muscle seemed surprising (Figure 1, Figure 2, Figure 3 and Figure S2). However, there is precedence in the literature.

(i) The *MLRN* RNA transcript was first identified as a functional lncRNA (*Linc*-*RAM*) that modulates myogenic differentiation in mice [114,115]. The *Linc*-*RAM* RNA was later found to serve also as a translated mRNA that encodes the 36-residue peptide MLRN that inhibits SERCA in mouse muscle [10,82]. Thus, it is possible that transcription and translation of SLN acts as key control mechanisms for the regulation of muscle development and contractility.

(ii) Human airway smooth muscle cells express abundant *PLN* RNA but do not express PLN protein [116]. Assays in this study included immunoblotting and immunoprecipitation of human airway smooth muscle homogenates, and immunohistochemistry of enzymatically isolated human airway smooth muscle myocytes. These antibody-based approaches were corroborated by functional assay of cultured human airway smooth muscle cells, whereby siRNA-PLN-knockdown had no effect on SR Ca^2+^ uptake in live cells or culture homogenates [116]. Similarly, our preliminary functional assays indicated that SLN protein is minimally expressed in horse gluteus muscle, since the anti-horse-SLN pAb 3379 has no effect on Ca^2+^ transport by SR vesicles from horse gluteal muscle, consistent with the lack of SLN protein expression (data not shown). These results indicate that the small level of SLN peptide expression detected in horse SR (~1 SLN molecule per 13–16 SERCA molecules) has a minimal effect on the regulation of SERCA activity in gluteal myocytes in vivo.

In addition, recent articles report transcriptional, translational, and protein degradation mechanisms whereby lncRNA regulate the expression of regulin-type inhibitory peptides and the activity of SERCA Ca^2+^ pumps in striated muscles. Here we describe newly proposed molecular mechanisms that may provide analogous insights into the high level of *SLN* gene expression and the low level of SLN peptide expression in horse muscle.

(i) The lncRNA *ZFAS1* in mouse heart is proposed to be a dual-mode inhibitor of SERCA that acts by (a) decreasing *ATP2A2* gene expression and (b) directly inhibiting SERCA2a enzyme activity [117]. Thus, *ZFAS1* lncRNA decreases the quantity and quality of Ca^2+^ transport in mouse SR. Analogously, we suggest the possibility that the high level of *SLN* RNA (sans SLN peptide) in horse gluteal muscle acts to regulate gene expression and/or activity of Ca^2+^ regulatory protein(s), including SERCA as a potential target. Indeed, *ZFAS1* lncRNA inhibits SERCA2a activity in vitro and in live cardiomyocytes, and over-expression of *ZFAS1* RNA in mouse left ventricle induces cardiac hypertrophy and ultimately heart failure [117].

(ii) The Veglia lab has demonstrated that random-sequence short RNAs, random-sequence single-stranded DNA oligonucleotides, and microRNAs #1 and #9 all bind the PLN peptide, thereby acting to relieve PLN inhibition of SERCA [118,119]. Thus, these recent studies indicate that nucleic acids show the potential to regulate a variety of central cellular functions through key molecular mechanisms, including Ca^2+^ regulation as a checkpoint hub. Indeed, there may be a species-specific array of currently unidentified lncRNA that act in distinct roles to regulate myofiber contractility and muscle adaptation in health and disease.

(iii) Hundreds of additional lncRNA with sORF (e.g., between 99 and 300 nucleotides) in human heart are reported to be translated as a peptide/small protein comprising 33 to 100 residues (also known colloquially as “micropeptide” or “microprotein”), including SR-targeted peptides that may act as regulators of cardiac contractility and development [120]. Thus, lncRNA may switch between functional modes: non-coding, or coding, or both.

### 4.5. Potential Mechanisms to Control SLN Protein Expression and SERCA Regulation in Horse Gluteal Muscle

In addition to the discussed possible mechanisms of direct control, there may be additional mechanisms by which the protein expression and targeting of the gene family of regulin-type peptides and SERCA proteins are controlled.

(i) Chaperone activity of SLN peptides in horse muscle. It is possible that SLN functions as a chaperone to target SERCA1 to longitudinal SR of skeletal muscle myofibers. For example, sAnk1 is peptide (non-regulin family member) that acts to help target SERCA1 to longitudinal SR and to maintain the ultrastructure of longitudinal SR of mouse myofibers [121]; in addition, sAnk1 also acts as a regulin-like peptide inhibitor of SERCA1 activity in a heterologous expression system [11,70]. On the other hand, the SERCA2 protein contributes to targeting PLN to longitudinal SR of cardiomyocytes, via the newly-identified NEST pathway (nuclear ER to SR via T-tubule transit), as reported by Cala and Chen [122,123]. Similarly, SERCA1 contributes to targeting SLN peptides to endoplasmic reticulum of cultured human embryonic kidney cells [35,36,124].

(ii) Degradation of SLN peptides in horse muscle. The peptide expression of PLN, the SLN-analog in ventricular and slow-twitch skeletal muscles, is controlled by multiple mechanisms. In the heart, PLN peptides are reported to be selectively degraded (relative to SERCA proteins) by a metformin-induced ubiquitin-mediated pathway and a phosphorylation-induced autophagy-mediated pathway [125,126]. In testes, PLN peptides are reported to be degraded by intramembrane proteolysis [108,127]. Thus, it is possible that horse muscle utilizes similar mechanism(s) for robust degradation of SLN peptides that are translated from the abundant level of SLN *RNA* transcripts.

(iii) RNA control of *SLN* transcription and translation in horse muscle. Micro-RNAs control transcription and translation of key targets (i.e., mRNAs, lncRNAs, and proteins) in numerous organisms and cell types [128,129,130]. Thus, it is possible that micro-RNAs in horse gluteal muscle act to shut down SLN peptide production from the high-level of *SLN* transcript production.

(iv) Control of SERCA protein degradation in horse muscle. The lncRNA *DACH1* is reported to directly bind and increase ubiquitination of SERCA2 in heart failure, thereby enhancing SERCA protein degradation, decreasing SR Ca^2+^ re-uptake, and inhibiting cardiac contractility [131]. It is possible that horse gluteal muscle has dynamically-regulated mechanisms to control the rate of SERCA1a degradation.

We propose that lncRNA help mediate the control of the steady-state level of cellular proteins that regulate Ca^2+^ homeostasis in horse gluteal muscle, using multiple mechanisms: transcription, translation, and degradation. Thoroughbred horses with recurrent exertional rhabdomyolysis (RER) have 50% faster relaxation times than horses that are not predisposed to recurrent exertional rhabdomyolysis [132]. It is possible that the defect in abnormal regulation of muscle contraction proposed to be the basis for recurrent exertional rhabdomyolysis arises from abnormal regulation of the steady state level of muscle proteins involved in Ca^2+^ regulation.

### 4.6. Study Limitations

Ideally, our transcriptomic analysis would have been performed in the same horses as SLN immunoblotting. We took advantage, however, of transcriptomic data already generated at considerable expense in Arabian horses, and we confirmed similar expression of *SLN*, *PLN*, *MRLN*, *DWORF,* and *ATP2A1* in the transcriptome of Warmblood horses (data not shown). Due to the large amount of muscle required to isolate a sufficient amount of SR vesicles to perform thorough biochemical analyses, euthanasia is required, and thus donated horses comprised the more common Quarter Horse and Thoroughbred breeds. We have also reported that the unique *SLN* sequence is identical across equine breeds such as Quarter Horse, Standardbred, Thoroughbred, Przewalski Horse, Zebra, and Donkey [32], therefore indicating that the breed differences of samples used in the present study did not have a major impact on our findings. Thus, we propose that quantitative comparison of gene and protein expression levels among different horse breeds is valid analytically.

## 5. Summary

The inhibitory functions of SLN on SERCA activity are clear *in vitro*. SLN gene expression and protein levels are increased many-fold in standard mouse models of Duchenne’s muscular dystrophy, e.g., *mdx* and *mdx*/*utr*-*dko* [111]. In compelling mouse and canine models of muscle disease, reducing SLN protein expression has been proposed to be an effective therapy [133]. However, decreasing SLN expression in alternative mouse models is reported to have beneficial or deleterious effects, depending on the genetic model and etiology studied [133,134,135]. To help delineate these disparate effects, additional correlations should be determined between Ca^2+^ transport regulation and muscular performance in large-animal models which may then provide insights into species-specific adaptations to enhance muscle contraction. Such studies could potentially impact therapeutic control of animal and human muscle disease. We suggest that further investigation is needed to define these pathways per species, muscle-type, and health status.

Our integrative transcriptional and biochemical methodologies provide novel information on gene and protein expression in horse SR, which is a unique physiological system for high-capacity Ca^2+^ transport. In horse gluteal muscle, we made two observations that were surprising: (i) SLN and PLN proteins were detected at miniscule levels in horse SR, i.e., SERCA is apparently unregulated by inhibitory transmembrane peptides in equine skeletal muscle, and (ii) the supra-abundant transcription of *SLN* mRNA is disconnected from stable expression of SLN protein. Data reported here contribute to the understanding of Ca^2+^ cycling in horse myofibers, with relevance to evolutionary development. Our ongoing functional studies continue to determine mechanistic roles of SERCA, SLN, CASQ, and RYR activities in horse muscle contractility, including the correlation of gene expression, protein homeostasis, and enzyme activity levels. We propose that experimental and computational analyses of the Ca^2+^ regulatory system in horse SR will enhance basic understanding of potential therapeutic targets to modulate muscle Ca^2+^ homeostasis.

## Supplementary Materials

The following two Supplementary Figures are available online at MDPI *Veterinary Sciences*, https://www.mdpi.com/2306-7381/7/4/178/s1; Figure S1: Immunoblot analysis using anti-human-SLN pAb 18395-1-AP detects minimal expression of SLN protein in horse and rabbit SR vesicles, and Figure S2: Immunoblot analysis using custom anti-rabbit/mouse/human-SLN pAb PFD-1 identifies SLN protein expression in rabbit SR vesicles, but not in horse SR.

## Acronyms and Abbreviations

*ATP2A1*, gene encoding SERCA1 protein isoforms; *ATP2A2*, gene encoding SERCA2 protein isoforms; *ATP2A3*, gene encoding SERCA3 protein isoforms; CASQ, calsequestrin; DWORF, dwarf open reading-frame peptide; *g*, gravitational force of 9.8 m/s^2^, defined at average Earth surface level; GP, glycogen phosphorylase; IU, international unit of enzyme activity, defined as the production of 1 µmol product per milligram protein per minute; K_Ca_, apparent Ca^2+^ dissociation constant, defined as the Ca^2+^ concentration required for half-maximal activation of SERCA activity; lncRNA, long non-coding RNA; mAb, monoclonal antibody; MRLN, myoregulin; pAb, polyclonal antibody; PLN, phospholamban; qRT-PCR, mRNA quantitation using real-time reverse transcription polymerase chain reaction; RER, recurrent exertional rhabdomyolysis; RNA-seq, whole transcriptome shotgun sequencing; RYR, ryanodine receptor Ca^2+^ release channel; SERCA, sarco/endoplasmic reticulum Ca^2+^-transporting ATPase; SLN, sarcolipin; sORF, small open reading frame; SR, sarcoplasmic reticulum; TPM, transcripts per million reads; V_max_, maximal enzyme velocity of SERCA, defined as Ca^2+^-activated ATPase activity at saturating concentration of ionized Ca^2+^ (~5 µM) and Mg•ATP (~5 mM).
