# Supplementary material for: Sarcolipin Exhibits Abundant RNA Transcription and Minimal Protein Expression in Horse Gluteal Muscle
Source: Vet Sci. 2020 Nov 13;7(4):178. doi: 10.3390/vetsci7040178 (PMC7711957; doi:10.3390/vetsci7040178)
Supplement: Supplementary file 1 [file vetsci-07-00178-s001.pdf]

SUPPLEMENTARY DATA

Sarcolipin exhibits abundant RNA transcription and minimal protein expression in horse gluteal muscle

Joseph M. Autry[1], Christine B. Karim[1], Sudeep Perumbakkam[2], Carrie J. Finno[3], Erica C. McKenzie[4], David D. Thomas[1,§], Stephanie J. Valberg[2,§]

Institutional affiliation:
[1]Department of Biochemistry, Molecular Biology, and Biophysics, University of Minnesota, Minneapolis, MN 55455
[2]Department of Large Animal Clinical Sciences, McPhail Equine Performance Center, Michigan State University, East Lansing, MI 48823
[3]Department of Population Health and Reproduction, University of California, Davis, CA 95616
[4]Department of Clinical Sciences, Oregon State University, Corvallis, OR 97331

[§]**Co-Senior Authors:** David D. Thomas and Stephanie J. Valberg

p. S1 **Table of Contents**

p. S2 **Figure S1**. Immunoblot analysis using anti-human-SLN pAb 18395-1-AP detects minimal expression of SLN protein in horse and rabbit SR vesicles.

p. S3 **Figure S2**. Immunoblot analysis using anti-rabbit/mouse/human-SLN pAb PFD-1 identifies SLN protein expression in rabbit SR vesicles, but not in horse SR.

p. S4 **References**

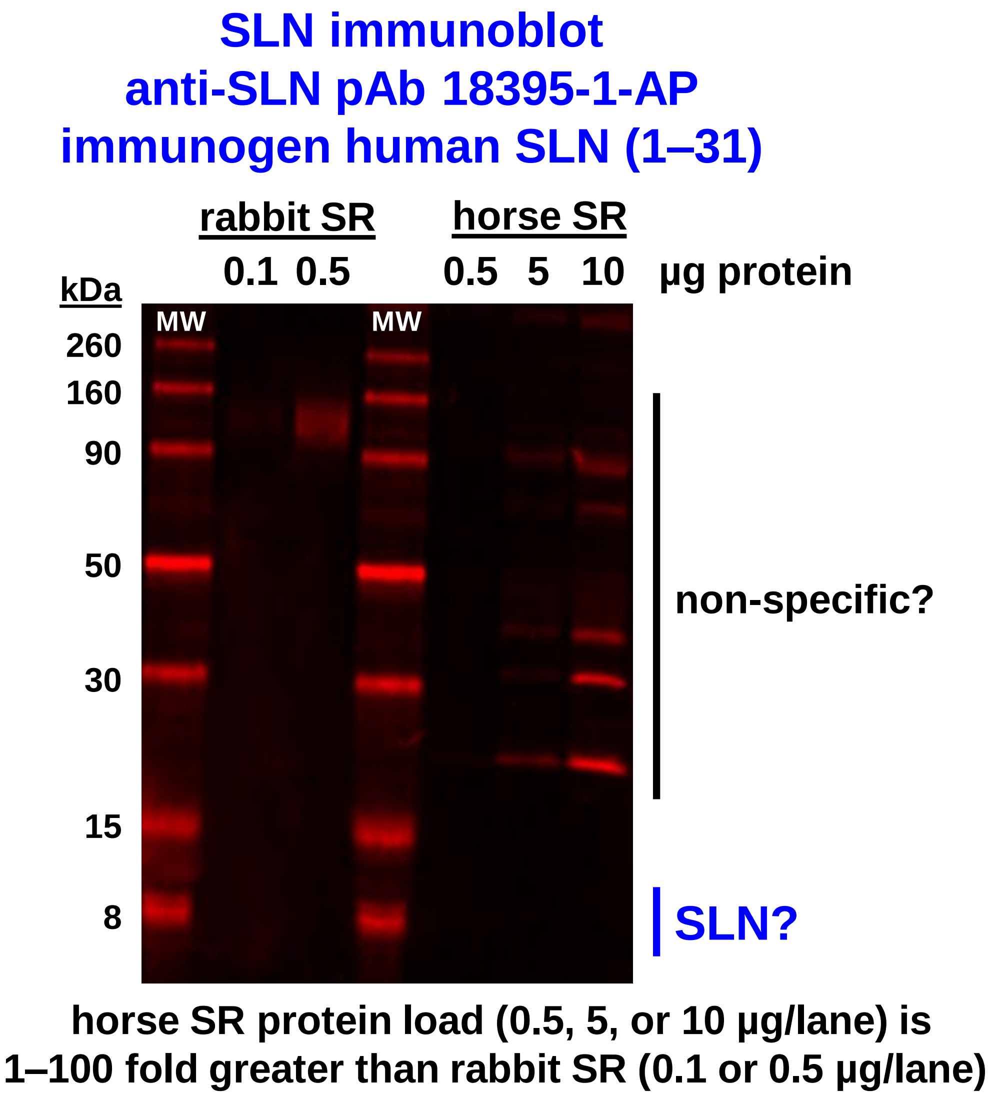

Figure S1. Immunoblot analysis using anti-human-SLN pAb 18395-1-AP detects minimal expression of SLN protein in horse and rabbit SR vesicles. The primary antibody was the commercial anti-SLN pAb18395-1-AP with immunogen comprising human SLN residues 1-31. SR samples were electrophoresed through a 4–20% Laemmli gel. Rabbit SR vesicles were loaded at 0.1 and 0.5 µg protein per lane (*left*), and horse SR vesicles were loaded at 0.5, 5, and 10 µg protein per lane (*right*). Immunoblotting was performed using pAb 18395-1-AP (primary) and goat anti-rabbit-IgG pAb labeled with 680-nm fluorophore (secondary). Immunolabeling was detected using a LI-COR laser scanner system in the near-infrared fluorescence mode. The molecular mass of protein gel markers (kDa) are indicated on the *left*.

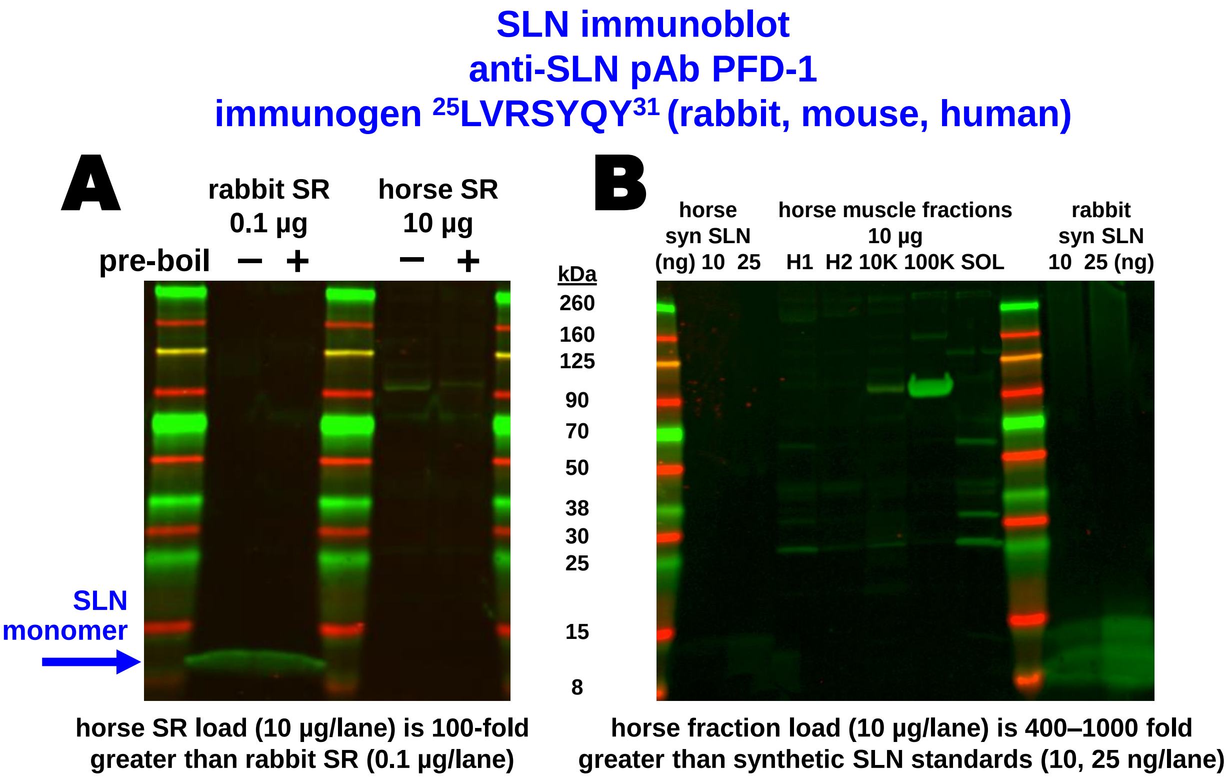

Figure S2. Immunoblot analysis using anti-rabbit/mouse/human-SLN pAb PFD-1 identifies SLN protein expression in rabbit SR vesicles, but not in horse SR. The primary antibody was the custom anti-SLN pAb PFD-1 [1] with immunogen comprising rabbit/mouse/human SLN residues ^{25}LVRSYQY^{31}, whereas horse SLN encodes ^{24}LVRSYQ^{29} [2]. Samples were electrophoresed through 4–20% Laemmli gels.). Immunoblotting was performed using pAb ABT13 (primary) and goat anti-rabbit-IgG pAb labeled with 680-nm fluorophore (secondary). Immunolabeling was quantified using a LI-COR laser scanner system in the near-infrared fluorescence mode. The molecular mass of protein gel markers (kDa) are indicated in the *middle*. (A) Rabbit SR vesicles were loaded at 0.1 µg protein per lane (*left*), and horse SR vesicles were loaded at 10 µg protein per lane (*right*). One sample of each SR set was heated at 100 °C for 2 min in Laemmli sample buffer prior to electrophoresis (+ pre-boil). (B) Synthetic SLN standard and horse muscle fractions.

REFERENCES

[1] P.F. Desmond, A. Labuza, J. Muriel, M.L. Markwardt, A.E. Mancini, M.A. Rizzo, R.J. Bloch, Interactions between small ankyrin 1 and sarcolipin coordinately regulate activity of the sarco(endo)plasmic reticulum Ca(2+)-ATPase (SERCA1), J Biol Chem 292 (2017) 10961-10972.
[2] S.J. Valberg, K. Soave, Z.J. Williams, S. Perumbakkam, M. Schott, C.J. Finno, J.L. Petersen, C. Fenger, J.M. Autry, D.D. Thomas, Coding sequences of sarcoplasmic reticulum calcium ATPase regulatory peptides and expression of calcium regulatory genes in recurrent exertional rhabdomyolysis, J Vet Intern Med 33 (2019) 933-941.
